# Serum Amyloid Alpha in Parapneumonic Effusions

**DOI:** 10.1155/2011/237638

**Published:** 2011-08-25

**Authors:** Vagelis Boultadakis, Vasilis Skouras, Demosthenes Makris, Aggeliki Damianaki, Dimitrios J. Nikoulis, Theodoros Kiropoulos, Smaragda Oikonomidi, Irene Tsilioni, Konstantinos Gourgoulianis

**Affiliations:** ^1^Respiratory Department, University Hospital of Larissa, Biopolis, 41110 Larissa, Greece; ^2^“Sismanoglio” General Hospital of Attica, 15126 Athens, Greece; ^3^Intensive Care Unit, University Hospital of Thessaly, Biopolis, 41110 Larissa, Greece; ^4^Respiratory Department, Chania General Hospital, 73300 Chania, Greece

## Abstract

*Study objectives*. To assess serum amyloid alpha (SAA) pleural fluid levels in parapneumonic effusion (PPE) and to investigate SAA diagnostic performance in PPE diagnosis and outcome. *Methods*. We studied prospectively 57 consecutive patients with PPE (empyema (EMP), complicated (CPE), and uncomplicated parapneumonic effusion (UPE)). SAA, CRP, TNF-*α*, IL-1*β*, and IL-6 levels were evaluated in serum and pleural fluid at baseline. Patients were followed for 6-months to detect pleural thickening/loculations. *Results*. Pleural SAA levels (mg/dL) median(IQR) were significantly higher in CPE compared to UPE (*P* < 0.04); CRP levels were higher in EMP and CPE compared to UPE (*P* < 0.01). There was no significant difference between IL-1*β*, IL-6, TNF-*α* level in different PPE forms. No significant association between SAA levels and 6-month outcome was found. At 6-months, patients with no evidence of loculations/thickening had significantly higher pleural fluid pH, glucose levels (*P* = 0.03), lower LDH (*P* = 0.005), IL-1*β* levels (*P* = 0.001) compared to patients who presented pleural loculations/thickening. *Conclusions*. SAA is increased in complicated PPE, and it might be useful as a biomarker for UPE and CPE diagnosis. SAA levels did not demonstrate considerable diagnostic performance in identifying patients who develop pleural thickening/loculations after a PPE.

## 1. Introduction

Parapneumonic effusions (PPE) affect 60.000 adults each year in the USA and UK and are associated with a morbidity of over 15% [1–4]. PPE may present in different forms, ranging from a self-resolving parapneumonic pleural effusion to complicated multiloculated and purulent effusions that may impair respiratory reserve. In this respect, diagnosis should be prompt especially in cases that drainage or/and more invasive type of management may be required.

Currently, management options for PPE are based on clinical and laboratory findings. Previous studies reported that pH, glucose, and lactate dehydrogenase (LDH) levels are strong indicators for the course of a PPE [5]. Other investigations reported that pleural fluid inflammatory cytokines which reflect the inflammatory and fibrotic processes in PPE such as TNF-*α*, IL-1*β*, and IL-6 can add useful information and may help in differentiating complicated parapneumonic effusions (CPEs) from uncomplicated parapneumonic effusions (UPEs) [6–8]. For example, the combined sensitivity of TNF-*α* and LDH to diagnose a complicated PPE is over 90% [9]. Other biomarkers which are associated with mechanisms implicated in the pathogenesis of pleural effusions—that is natriuretic peptides, SAA—have been also suggested to aim in the diagnosis of pleural effusions [10]. However, data for the role of these indices in PPE are sparse, and their potential role in the diagnosis and outcome of PPE is not clear. 

In the present prospective study, we aimed to investigate prospectively the role of SAA in the diagnosis and prognosis of complicated effusions.

## 2. Materials and Methods

Patients were recruited by consecutive sampling from the emergency departments at the University Hospital of Larisa and “Sismanoglio” General Hospital of Attica between January 2007 and January 2008. Patients were included in the study when they fulfilled the following criteria: (i) pleural effusion characterized as exudates according to criteria suggested by Light [5], (ii) predominance of neutrophils cells in the pleural fluid, (iii) diagnosis of pneumonia based on ATS criteria, (iv) no antibiotics for PPE prior to admission. Patients treated with local fibrinolytics were excluded from the study. The study was approved by the Local Ethical Committee, and all patients gave their consent to participate in the study. 

Previously accepted criteria were used for the definition of PPE as Empyema (EMP) or, complicated parapneumonic effusion (CPE) or, uncomplicated parapneumonic effusion (UPE) [5, 11, 12]. EMP included grossly purulent PPE and nonpurulent PPE if microorganisms were detected by Gram-stained smears or by pleural fluid cultures [12]. CPE included nonpurulent effusions with pleural fluid pH level <7.2 or, pleural fluid glucose <40 mg/dL or, pleural fluid LDH level >1000 U/L [11] or, when there were apparent loculations on the chest X-ray or ultrasound examination of the chest. Effusions were grouped as UPE if none of the above properties were present [11]. 

At baseline, patients underwent clinical and radiologic assessment and were followed for up to 6 months. Treatment of all cases was based on previously accepted guidelines, [13] and fibrinolytics were not used. Patients underwent clinical and radiological evaluation at 6 months and were classified according to the presence of loculations/pleural thickening/respiratory impairment or not.

### 2.1. Biochemistry and Cytology of the Fluid

Pleural fluid samples were obtained at baseline by thoracentesis before the institution of any antibiotic treatment and were immediately analyzed for pH (Instrumentation Laboratory, USA). Total cell count, differential cell count, total protein, glucose, and LDH were measured in pleural fluid and serum. Pleural fluid samples were centrifuged at 1500 g for 15 minutes, and the supernatant from each sample was stored at −80°C to measure Il-1*β*, Il-6, TNF-*α* and SAA and CRP.

### 2.2. Cytokine, SAA, and CRP Assays

SAA and CRP were measured in serum and pleural fluid using nephelometry (Behring Nephelometer Analyzer II) using the N High Sensitivity kit (Dade Behring, Marburg, Germany). Il-1b, Il-6, and TNF-*α* in supernatant of the samples were measured by using an immunoenzymometric assay (Biosource Inc; Europe S.A.). The reproducibility of these assays was confirmed by performing repeated measurements on successive days.

### 2.3. Chest Ultrasound and Radiographic Assessment

Patients underwent chest X-ray and ultrasound of the chest using a linear transducer (Aloka Echo Camera SSD—650^CL^) for evaluation of (a) the distance between parietal and visceral pleura, (b) the presence of septa/loculations or not, and (c) the degree of septal thickening (mm). PPE were classified as anechoic when echo-free spaces were present between the visceral and parietal pleura, as echogenic when an internal sonographic pattern was present and echogenic septated.

### 2.4. Statistical Analysis

Descriptive statistics were used to summarize the baseline characteristics, and the results were expressed as means (SE) or stated otherwise. Normal distribution was assessed using the Kolmogorov-Smirnov *Z* test. ANOVA (with Bonferoni's test for comparison between groups) was applied for the comparison of continuous variables. Categorical variables were compared using the chi-square test. To determine the prognostic value of various parameters in predicting favourable or not outcome, receiver operating characteristic (ROC) curves were constructed to assign cutoff values and their diagnostic utility. A *P* value of less than 0.05 was considered as statistically significant. The statistical package SPSS 13.0 (Chicago, Ill, USA) was used for the entire analysis.

## 3. Results

The study population comprised 57 patients (39 men and 18 women), with median age of 67.5 (57.25–71.75) years. Baseline characteristics of participants are presented in Table 1. Twenty-six patients had UPE, 17 patients CPE, and 14 patients had EMP. At six months, 8 (14%) patients died, 8 (14%) presented evident loculations, and 5(8.7%) pleural thickening >1 mm. Thirty-six out of 57 patients (61.4%) had a favorable course on the basis of an uneventful clinical course and absence of loculations or pleural thickening.

### 3.1. Pleural Fluid SAA, CRP, TNF-a, IL1*β*, IL6

Pleural fluid characteristics are presented in Table 2. Total white cell counts (cells/*μ*L ×10^3^) median (IQR) were significantly lower in UPE [7300 (2340–10950) *P* < 0.009] and CPE [2600 (850–5600), *P* < 0.015] compared to EMP. SAA pleural fluid levels (mg/dL) were significantly higher in CPE [12.35 (2.6–30.8)] compared to UPE [6.2 (2.4–15.07)], (ANOVA, *P* < 0.04). CRP levels (mg/dL) were significantly higher in Empyema [10 (4.1–11.8)] and CPE [12.2 (7.85–14.42)] compared to UPE [4.3 (1.9–5.82)] (*P* < 0.01). There were no statistically significant differences in terms of TNF-*α*, IL-1*β*, and IL-6 between groups.

### 3.2. Serum SAA, CRP, TNF-*α*, IL1*β*, IL6

Serum levels of biochemical parameters and of different cytokines are shown in Table 3. Median (IQR) SAA serum levels were found higher in CPE [80.95 (31.73–98.575)] compared to UPE [29.25 (7.525–74.225) (*P* = 0.079)] and EMP [52.2 (28.7–62.4) (*P* = 0.579)], but there was no statistical significance. CRP was significantly higher in CPE [18.75 (15.4–20.4)] than UPE [7.25 (4–16.85), *P* < 0.011]. There were no differences in terms of TNF-*α*, IL-1*β*, and IL-6 between groups. 

### 3.3. Relationship between Inflammatory Markers and PPE Outcome at 6 Months

Patients with favorable outcome (uneventful clinical course, absence of loculations or pleural thickening) had significantly higher pleural fluid pH and glucose levels (*P* = 0.03 and *P* = 0.03, resp.) and lower LDH levels (*P* = 0.005) compared to patients who presented loculations/pleural thickening at 6 months (Table 4). In addition, IL-1*β* was significantly higher in the pleural fluid of patients with loculations/pleural thickening [13.33 (10.15–30.44)] compared to patients with favorable outcome [2.01 (0.80–11.89) (*P* < 0.05)]. No significant differences were found in terms of SAA, CRP, TNF-*α*, IL-6 levels in pleural fluid. 

**Figure 1:**
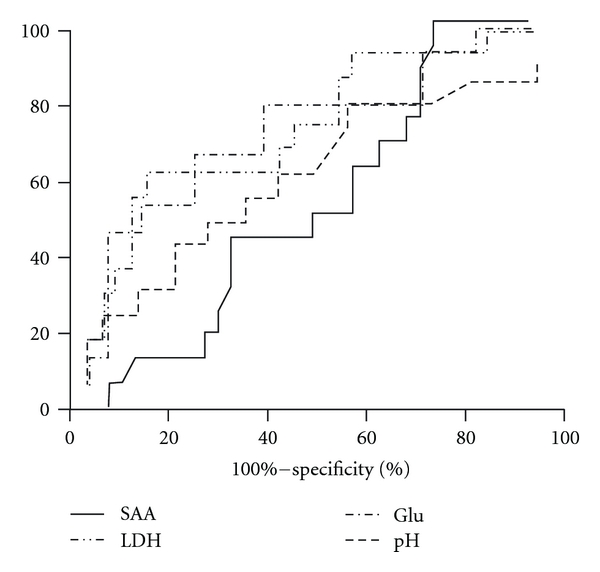
ROC curves for SAA, Glucose, pH, LDH in identifying PPE patients with favourable course and outcome at 6 months.

ROC analysis evaluated the diagnostic performance of inflammatory pleural fluid markers in the diagnosis of outcome at 6 months. AUC for ph, glucose, LDH were 0.65, 0.73, and 0.72, respectively (Figure 1). A cutoff point of >120 mg/dL pleural glucose had 100% specificity for the diagnosis of favourable outcome; accordingly, the cut-off point for LDH was <160 mg/dL.

## 4. Discussion

In the present study we found that SAA levels in pleural fluid were significantly higher in patients with CPE compared to UPE. This finding suggests that SAA might play a role in the inflammatory process that characterizes parapneumonic effusions. However, our findings did not support the hypothesis that SAA might have also a role in the prediction of clinical course of PPE. Despite that several biochemical markers of inflammation such as pleural pH, glucose, LDH, and CRP were significantly associated with 6-month outcome, SAA did not present significant diagnostic performance in identifying patients who would present pleural thickening/loculations or die at 6 months following admission for PPE. 

The role of SAA in PPE is not well known. Only one study conducted by Okino et al. [10] investigated the role of SAA in patients with PPE. The authors compared pleural SAA, CRP, and pleural fluid/serum ratios and pointed out SAA as a very good marker in discriminating between exudates and transudates. In agreement with that study [10], SAA pleural fluid levels (mg/dL) in the present investigation were significantly higher in CPE compared to UPE (*P* < 0.04). However in the present study we followed patients for 6 months, and we provided further data regarding the relationship between SAA and the clinical, radiologic outcome of patients with PPE. SAA failed to show a significant association with the outcome of PPE. In contrast, parameters which have been previously related to prognosis and have been widely used for management decisions [5] presented remarkable diagnostic performance. Notably, the diagnostic performances of pleural glucose and LDH in identifying patients with favourable outcome at 6 months were considerable; pleural glucose value of >120 mg/dL or LDH of <160 mg/dL had 100% specificity for the diagnosis of favourable outcome. In this respect, our results suggest that SAA could be used at least, as an alternative marker for discriminating between CPE and UPE. 

SAA is a protein which is usually produced in the acute phase of inflammation-as CRP-and in this respect the finding of elevated levels in PPE might not be surprising [14–16]. However, SAA levels were significantly higher in patients with complicated effusions. In this respect, increased SAA levels might represent the burden of inflammation in the pleura. On the other hand, SAA levels in EMP were low—notable are the levels that were similar to uncomplicated effusions. A plausible explanation for this might be the fact that SAA is a protein that can be easily degraded in the purulent environment of EMP where neutrophils predominate [17]. 

SAA predominantly produced and secreted mainly by hepatocytes and by other cells including lymphocytes, monocytes, and macrophages [14, 15]. Induction of SAA synthesis is triggered by a number of cytokines, chiefly IL-6, which is released from a variety of cell types, but mainly from macrophages and monocytes at inflammatory sites [16]. TNF-*α* and IL-1*β* may also act as inflammatory mediators that induce SAA [18]. In the present study, we therefore, measured levels of these cytokines in the pleura fluid and serum. We found no significant correlation between SAA levels and TNF-a, IL-6, or IL-1*β*. However, this does not exclude any relationship between SAA and TNF-a, IL-6 or IL-1*β* because these cytokines are widely involved in the inflammatory cascade, might be affected by many factors and could be upregulated in PPE regardless of SAA levels. TNF-a, IL-6, or IL-1*β* regulate the growth and differentiation of a variety of immune cells and play role in a variety of inflammatory reactions. Notably, IL-6 is often used as a marker of systemic activation of proinflammatory cytokines [19]. Increased levels of TNF-*α* have been found in various infections and in parapneumonic effusions [9, 20]. IL-1*β* has various biologic activities, principally as an immunomodulator and proinflammatory mediator per se or via induction of other cytokines and inflammatory mediators [21]. 

On this basis the levels of these cytokines vary in different studies where different populations were included and different techniques were used. Thus, Silva-Mejias et al. showed that IL-1*β* levels were >200 pg/mL in patients with parapneumonic effusions [22], but in another study [23] levels in infectious and neoplastic etiologies were similar. In our study, there was a trend towards higher IL-1*β* levels in CPE compared to UPE (*P* < 0.088). In this respect, universally accepted cut-off diagnostic values in the case of PPE are hard to be applied. However, these cytokines may reflect the burden of the systemic inflammation or the inflammatory process in the pleura and might provide useful information. Notably, in our study, IL-1*β* was associated with the outcome at 6-month. 

In conclusion, our findings suggest that SAA is increased in complicated PPE, and it might be used as an alternative biomarker in the diagnosis of nonpurulent complicated parapneumonic effusions. However, SAA levels did not demonstrate considerable performance in identifying patients who develop pleural thickening or loculations after a PPE in our study, whereas classic parameters such as pH, glucose, and LDH did. In this respect, based on the current clinical management of PPE [24], the present study cannot advocate the use of SAA in the routine practice.

## Figures and Tables

**Table 1:** Baseline characteristics of all 57 participants.

Age	67.5 (57.25–71.75)
SpO2	95 (92.5–95.5)
CURB score	1 (1-2)
Comorbidities- Carlson index, *n *	1 (0–2)
Presence of chronic respiratory disease, *n *	18
Loculations in ultrasound, *n *	24
Septal thickening in ultrasound (mm)	3 (2-3)
Pleural effusion extended in	
>half hemithorax, *n *	9
>1/3	16
<1/3	18

Continuous data are expressed as median (IQR), otherwise indicated.

**Table 2:** Pleural fluid characteristics in different PPE types.

	EMP (*n* = 14)	CPE (*n* = 17)	UPE (*n* = 26)
Total cell count, cells/*μ*L×10^3 ∗∗, ##, *∧*^	32 (9–109.5)	2.6 (0.85–5.6)	7.3 (2.34–10.95)
* Neutrophils, %*	80 (68–90)	83 (62.75–90)	73 (40–83)
Albumin g/dL^∗, ##^	2.2 (1,75–2.9)	2.55 (1.775–3.05)	3.28 (3.32–4,68)
Protein, g/dL	4.65 (3.06–4.97)	4.71 (4.167–5.54)	4.35 (2.972–5.13)
Glucose, mg/dL^∗∗, ##^	6 (1.85–46)	23 (5.45–58.25)	102 (90.5–125.7)
LDH, U/L^∗∗, ##^	6035 (3138–10085)	2113 (1350–3521)	446.5 (332–575)
ph^∗∗, ##^	6.768 (6.07–7.13)	6.92 (6.77–7.10)	7.409 (7.3727.45)
SAA, mg/dL^∗, *∧*^	3.2 (0.6–8.9)	12.35 (2.6–30.8)	6.2 (2.4–15.07)
CRP, mg/dL^∗∗, ##^	10 (4.1–11.8)	12.2 (7.85–14.42)	4.3 (1.9–5.82)
TNF-*α*, pg/mL	37.77 (13.45–55)	34.06 (22.57–101.79)	16.746 (9.456–42.59)
IL-1*β*, pg/mL	11.74 (3.15–26.52)	2.02 (0.88–5.30)	0.7485 (0.175–9.39)
IL-6, pg/mL	50.622 (27.45–73.8)	95.36 (76.93–197.78)	323.75 (1.83–398.55)

Data are presented as median (IQR). *P < 0.05 and **P < 0.01 between CPE and UPE. ^#^ *P* < 0.05 and ^##^ *P* < 0.01 between UPE and EMP. ^*∧*^ *P* < 0.05 and ^*∧∧*^ *P* < 0.01 between CPE and EMP. EMP: Empyema, CPE: Complicated Parapneumonic Effusions, UPE: Uncomplicated Parapneumonic Effusions.

**Table 3:** Serum levels of biochemical parameters and of different cytokines in PPE.

	EMP (*n* = 14)	CPE (*n* = 17)	UPE (*n* = 26)
Glucose, mg/dL	124 (102–182.5)	97 (86–141)	101 (88–120)
LDH, U/L	288 (202–325)	266 (259–301)	264.5 (234.25–286.75)
pH	7.465 (7.443–7.5)	7.47 (7.445–7.485)	7.47 (7.445–7.488)
SAA, mg/dL	52.2 (28.7.–62.4)	80.95 (31.73–98.575)	29.25 (7.525–74.225)
CRP, mg/dL*	11.9 (10–19)	18.75 (15.4–20.4)	7.25 (4–16.85)
TNF-*α* pg/mL	10.24 (7.783–12.55)	14.96 (8.1–19.44)	13.42 (5.86–18.29)
IL-1*β* pg/mL	0.9 (0.79–1.065)	0.66 (0.4475–1.66)	1.365 (0.6725–13.39)
IL-6 pg/mL	44.69 (26.17–56.99)	56.52 (36.75–83.44)	64 (16.35–101.11)

Data are presented as median (IQR). *P < 0.01 between CPE and UPE. EMP: Empyema, CPE: Complicated Parapneumonic Effusions, UPE: Uncomplicated Parapneumonic Effusions.

**Table 4:** Pleural fluid characteristics in PPE according to outcome at 6 months.

	Favourable outcome (*n* = 36)	Loculation/pleural thickening (*n* = 13)	Died (*n* = 8)
Total cell count, cells/*μ*L ×10^3^	7.2 (4.2–12)	4.37 (9.06–182.5)	2 (1.09–13.84)
Protein, g/dL	4.6 (3.75–5.27)	4.35 (2.945–4.8225)	4.3 (2.69–4.8)
Glucose, mg/dL*	89 (56.45–112.75)	5.5 (1.52–67)	60 (13.5–83.5)
LDH, U/L+**	603 (340.25–1655)	6641 (2334–9828)	625 (463.5–9646)
pH**	7.34 (6.95–7.43)	6.81 (6.21–7.06)	7.35 (7.03–7.41)
SAA, mg/dL	6.7 (2.55–13.3)	7.05 (1.27–16.87)	1.05 (0.4–4.62)
CRP, mg/dL	5.2 (2.5–11.55)	7.4 (4.07–11.72)	4.6 (2.72–7.59)
TNF-*α*, pg/mL	25.17 (11.77–59.73)	41.15 (6.67–57.69)	22.95 (12.31–43.45)
IL-1*β*, pg/mL*	2.01 (0.80–11.89)	13.33 (10.15–30.44)	0.56 (0.162–25.54)
IL-6, pg/mL	341.83 (146.56–383.47)	2.456	4.279 (2.6415–239.111)

Data are presented as median (IQR).

*P < 0.05 and **P < 0.01 between favorable outcome and loculations.
